# Coal Combustion Wastes Reuse in Low Energy Artificial Aggregates Manufacturing

**DOI:** 10.3390/ma6115000

**Published:** 2013-10-31

**Authors:** Claudio Ferone, Francesco Colangelo, Francesco Messina, Fabio Iucolano, Barbara Liguori, Raffaele Cioffi

**Affiliations:** 1 INSTM Research Group Naples Parthenope, Department of Engineering, Parthenope University of Naples, Centro Direzionale Naples, Isola C4, Naples 80143, Italy; E-Mails: francesco.colangelo@uniparthenope.it (F.C.); francesco.messina@uniparthenope.it (F.M.); raffaele.cioffi@uniparthenope.it (R.C.); 2 Department of Chemical, Materials and Production Engineering, University of Naples Federico II, Piazzale Tecchio, 80, Naples 80125, Italy; E-Mails: fabio.iucolano@unina.it (F.I.); barbara.liguori@unina.it (B.L.)

**Keywords:** sustainable concrete and mortars, artificial aggregates, weathered coal fly ash, wastewater treatment sludge, desulfurization device sludge

## Abstract

Sustainable building material design relies mostly on energy saving processes, decrease of raw materials consumption, and increase of waste and by-products recycling. Natural and lightweight artificial aggregates production implies relevant environmental impact. This paper addresses both the issues of residues recycling and energy optimization. Particularly, three coal combustion wastes (Weathered Fly Ash, WFA; Wastewater Treatment Sludge, WTS; Desulfurization Device Sludge, DDS) supplied by the Italian electric utility company (ENEL) have been employed in the manufacture of cold bonded artificial aggregates. Previously, the residues have been characterized in terms of chemical and mineralogical compositions, water content, particle size distribution, and heavy metal release behavior. These wastes have been used in the mix design of binding systems with the only addition of lime. Finally, the artificial aggregates have been submitted to physical, mechanical, and leaching testing, revealing that they are potentially suitable for many civil engineering applications.

## 1. Introduction

### 1.1. Research Significance

Many authors have already discussed the environmental impact of building material industry [1,2,3]. In this context, concrete and cementitious composites found a field of tremendous growth, so that, nowadays, concrete is the most used material throughout the world. The analysis of environmental issues associated with the construction industry must be carried out, considering just one final application because every material has particular production process parameters (raw materials supplying/processing, casting/curing conditions, *etc.*). When the whole engineering application is defined, the user can assess what is the design strategy that has to be followed in order to increase the sustainability. Generally, the designer/analyst will act on one or more of the following issues:

1. Energy saving in production processes;
2. Decrease of natural raw materials consumption;
3. Recycling of wastes/by-products of other industrial processes.

**Figure 1:**
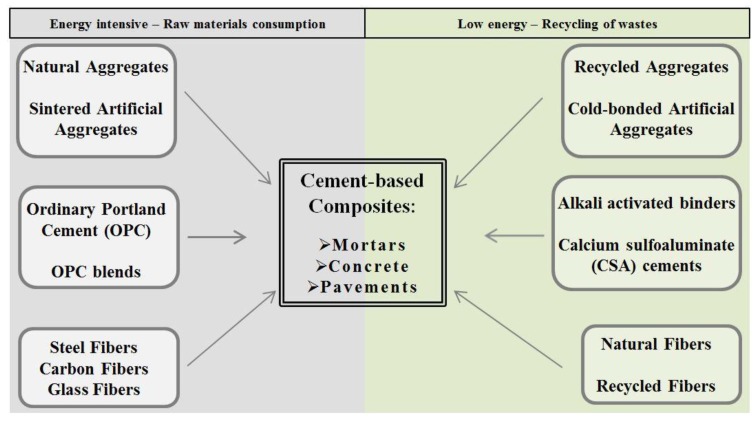
Components of cementitious composites in a sustainable design scheme.

In cementitious binding systems, excluding chemical admixtures, it is possible to find different components, depending on the particular structural aim of the design (reinforced concrete for residential buildings, precast concrete for bridges, concrete for pavements, rendering mortars, *etc.*). A brief review is reported in Figure 1.

In Figure 1 it is possible to distinguish three different lines of action for the sustainable design practice, each one corresponding to a component of the composite: (1) aggregates, both natural and artificial; (2) binding systems, which are extremely variable depending on the application; and (3) fibers, not always employed, which are used in fiber reinforced concrete, mortars, *etc.* Furthermore, two opposite sides (the first one in grey, the second one in green) collect environmentally hostile and friendly options for materials design in civil engineering. Even if sustainability is a broader concept, also contemplating social and economic issues, the above-described approach can help designers/analysts in the initial phases of a project, where few variables can be taken into account, depending above all on the construction site/area. In the first action line (aggregates), the introduction in the market of recycled and artificial cold bonded aggregates is highly desirable, both for natural resources depletion reduction, and wastes reuse, with potential market in structural and environmental technologies [4,5,6,7,8,9,10,11]. In the second one (binding systems), ordinary Portland cement (OPC) and OPC blends still play a key role. Greener options are, thus, needed in order to reduce the environmental issues related to relevant amounts of CO_2_ release in the atmosphere by the clinker synthesis process. Even if few real applications can be found, a huge number of experimental studies are available in literature. Developed at the end of the last century, calcium sulfoaluminate (CSA) cements represent one of the first attempts in the reduction of clinker impact on the environment [12,13]. More recently, research attention on alkali activated binders is relevantly increased, frequently presented as a green alternative to traditional cements, both for low process and curing temperatures and for the possibility of employing wastes/by-products/sludge as raw aluminosilicate powdered precursor [14,15,16,17,18,19]. This new class of materials has been widely tested for several civil and environmental engineering applications, but there is still a lack of data on the behavior of these materials under service life conditions [20,21,22,23,24,25,26]. In the case of fiber reinforcements (action line 3) able to give composites with improved physico-mechanical performances, natural and recycled fibers are of great interest [27,28].

This paper focuses on the possibility of employing coal combustion wastes in a cold bonding pelletization process in order to produce sustainable artificial aggregates, developing, thus, a possible alternative in the above described action line 2. The possibility of employing residues with binding capability or filler physical properties is typical of a cold bonding pelletization process [11,13,16,17]. In fact, in order to obtain aggregates with sufficient mechanical properties, both binding and filler agents are needed. The environmental benefits of this process do not rely just on materials recycling, but also on the very low temperature at which the granulation is carried out. In fact, the cold bonding pelletization is usually carried out at room temperature.

### 1.2. Coal Combustion Residues Environmental Issues and Recycling Strategy

Coal fired power plants are still one of the main electrical energy sources throughout the world. Despite new designs, they are responsible of relevant greenhouse gases emissions, contributing to both air pollution and acid rain [29].

**Figure 2:**
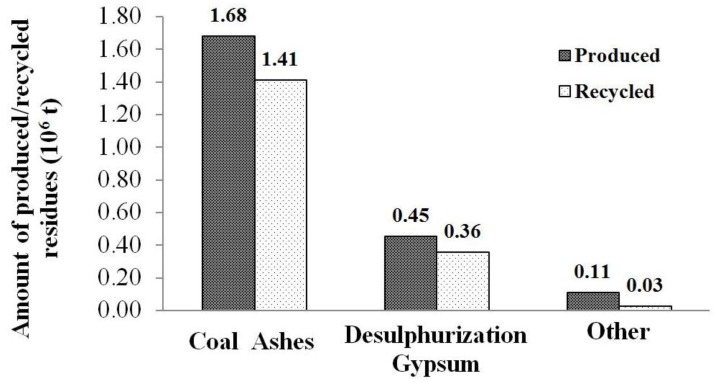
Coal combustion residues amounts production and recycling in Italy in 2012 (adapted with permission from ENEL Environmental Report, 2012 [30]. Copyright by the National Agency for Electric Energy, ENEL).

According to the Italian electric utility company (ENEL, Rome, Italy,) [30], in 2012, forty-three thermoelectric power plants are operating in Italy, with a net power, which can be estimated to be about 24,723 MW. Many different fossil fuel resources are used and, particularly, coal represents the first one, covering 75.41% of the needs. These combustion-based energy production processes generate many industrial outputs. First of all, the total amount of equivalent CO_2_ is equal to about 38 × 10^6^ t. Furthermore, both liquid and solid residues are produced. Wastewater quantity estimation is about 6 × 10^6^ m^3^ and approximately 88% of this is reused inside the plants. As far as solid wastes are concerned, about 2.3 × 10^6^ t of special wastes have been produced in 2012, and 80% of these have been recycled. Particularly, 99% of the special wastes is made of non-hazardous wastes. The specific production of coal combustion residues is reported in Figure 2 [30].

The major impacts of coal combustion solid residues disposal on terrestrial ecosystems include mostly the leaching of potentially toxic substances into soils and groundwater and several potential attacks to vegetation (reductions in plant establishment and growth; changes in the elemental composition of vegetation; increased mobility and accumulation of potentially toxic elements throughout the food chain) [29].

This study deals with a recycling strategy of coal combustion residues for building materials industry. Particularly, three residues coming from Brindisi (Italy) ENEL power plants have been considered: Weathered Fly Ash (WFA), Wastewater Treatment Sludge (WTS), and Desulfurization Device Sludge (DDS). These residues have been previously characterized in terms of physical and chemical properties. In the second phase of experimental program mixtures of the artificial aggregates have been designed and the setting of the cold bonding process has been fixed. In the last phase, the produced artificial aggregates have been submitted to physical and mechanical testing. The heavy metals leaching behavior of the cementitious mixtures have been also evaluated.

The cold bonded aggregates exhibited good physical and mechanical properties, and the WFA/lime binding matrix showed a relevant capability to stabilize heavy metals.

## 2. Experimental Program

### 2.1. Coal Combustion Residues Physical and Chemical Characterization

WFA stands for fly ash coming from wet storage, without drying. In this case, fly ash comes from a temporary storage basin of an ENEL coal-fired power plant, located in Brindisi (Italy). The ash is periodically sprinkled with water and so resulting waste is of a moist consistency with water content much lower than that would be given by pond storage. This waste must be distinguished from rejected fly ash, a low-grade fly ash, which is not used in blended cements due to its high carbon content and particle size mostly higher than 75 µm [31]. Particle size distribution of WFA has been determined by means of a Malvern Instrument Mastersizer 2000 laser scattering analyzer (Worcestershire, UK, particles observation range: 0.02 ÷ 2000 µm), after that the ash has been dried in an oven at 105 °C, without grinding operations. For coarse samples, namely WTS and DDS, particle size analysis has been carried out by means of mechanical sieving, according to the UNI EN 933-1 and UNI EN 933-2 standards [32,33]. After this, the two wastes have been grinded using a Retsch^®^ DM 200 disc mill (Haan, Germany, material feed size < 20 mm, final fineness < 100 µm).

Water content of the residues has been obtained after drying the samples in an oven at 105 °C in the case of WFA, and 60 °C in the other cases. Loss on ignition (LOI) has been determined by thermal heating up to 1050 °C. The three wastes have been submitted to total acid digestion (ASTM 5258-92) [34] and subsequent chemical analysis through inductively coupled plasma atomic emission spectrometry (ICP-AES) technique for the determination of metals content. The mineralogical analysis (X-ray diffraction analysis, XRD) has been performed by means of a Philips PW 1730 diffractometer (Amsterdam, the Netherlands, Cu Kα radiation, 2θ = 5°–60° , step width 2θ = 0.02° and 1 s data collection per step).Thermal analysis has been carried out by means of thermogravimetric analysis (TGA), making use of a TA Instruments thermal analyser SDT2960 (New Castle, USA) (weight of the sample: 10 mg; heating rate: 10 °C∙min^−1^; atmosphere: air).Finally, the residues have been submitted to UNI EN 12457-2 [35] leaching test. This is a static test that makes use of deionized water as leachant in a 10 L∙kg^−1^ liquid to solid ratio. Its duration, in the case of granular solids (size < 3 mm), must be 24 h. To prevent metals precipitation after leaching, the leachate pH must be brought to 2 by means of 1 N HNO_3_ solution.

### 2.2. Artificial Aggregates Production and Testing

The manufacture of cold bonded aggregates has been carried out by means of a laboratory-scale pelletization apparatus. Process parameters have been chosen on the basis of previous observations, which can be found in literature [11,13,16,17]. The pelletization apparatus is equipped with an 80 cm diameter rotating disk whose tilting angle has been fixed at 50°. The revolution speed was fixed at 45 rpm. The powders have been previously mixed with water by means of a Hobart mixer and then slowly and continuously poured onto the disk. The water to solid ratio has been adjusted during the production process using a nozzle to spray more water into the mixes. Mix design of aggregates is reported in Table 1. In all designed mixtures an amount of lime ranging from 10% to 40% has been added to the wastes. The set of compositions is essentially made of three subcategories. The first one is represented by C*_x_* mixtures, where *x* is the WFA content (wt. %). Among these three mixtures, the reference is C_60_ where the WFA/L ratio is fixed at 3:2 in order to obtain the maximum amount of cementitious hydration products, on the basis of the well-known activating capability of Ca(OH)_2_. The other two mixes (C_80_ and C_90_) have been studied to maximize the amount of reused waste. The second subcategory of compositions is the C*_x_*W*_y_* one, where x is again WFA content, while y stands for WTS content. C_30_W_50_ and C_24_W_60_ compositions represent trials to reuse WTS in a C–S–H matrix obtained by keeping WFA/L ratio at 3:2. A narrow range of WTS content has been chosen because of the decay of the physico-mechanical performance, due to the unreactive nature of this waste. On the other side a low amount of waste would reduce the benefit of the recovery process. Finally, the C*_x_*D*_y_* compositions have been designed on the basis of previous experience [36], where a tuff-lime-gypsum system was studied. WFA:L:DDS reference ratio has been fixed at 4:3:3 because, according to the above mentioned study, this mixture is theoretically able to give the conversion of 35% of silica into C–S–H and 70% alumina into ettringite.

Curing conditions have been chosen in order to obtain sufficient mechanical performance without increasing the energy consumption of the process. For this reason, artificial aggregates were cured for 56 days at room temperature and 100% RH, and experimental results were simultaneously compared to those obtained by curing the specimens at 40 °C for the first three days. At the end of the curing phase the grain size distribution of the hardened products has been determined by sieving, according to [32].

**Table 1:** Artificial aggregates compositions, wt %.

Mixtures	WFA	L	DDS	WTS	W/S ratio
C_90_	90	10	–	–	0.22
C_80_	80	20	–	–	0.25
C_60_	60	40	–	–	0.29
C_30_W_50_	30	20	–	50	0.25
C_24_W_60_	24	16	–	60	0.30
C_40_D_30_	40	30	30	–	0.25
C_25_D_10_	25	15	10	50	0.28
C_20_D_15_	20	15	15	50	0.31

In order to evaluate hydration kinetics of the most complex system, which is represented by C*_x_*D*_y_*, differential thermal analysis (DTA) has been performed on C_40_D_30_ where WTS has not been employed. This analysis has been carried out on samples cured at room temperature and after 40 °C curing. The instrument used for this test was the same used for residues characterization.

Successively, the artificial aggregates have been submitted to the following tests: dry density and water absorption capacity, crushing strength, and Los Angeles coefficient. Water absorption was determined according to the UNI EN 1097-6 standard [37]. Samples were first dried in the oven at 110 ± 5 °C until constant mass. The measured values of dry mass (*m*_d_) and saturated surface-dry mass (*m*_ssd_) after 24 h immersion were used in the calculation of water absorption (WA):

(1) $$\text{WA}=\;\frac{m_{\text{ssd}}-m_{\text{d}}}{m_{\text{d}}}\;100$$

Crushing tests were performed, according to the UNI EN 13055-1 standard [38], in order to determine aggregates engineering properties. The aggregates were placed in a steel cylinder and compacted by means of mechanical vibration. After that, samples were charged through a steel piston by means of a CONTROLS^®^ 50-C56Z00 compression testing machine [Cernusco (MI), Italy] with a capacity of 3000 kN. The test method for the Los Angeles Coefficient involves a test aggregate sample with particles between 10 mm and 14 mm in size. The sample is rotated in a steel drum with a specified quantity of steel balls, at a speed of 31 to 33 revolutions per minute for 500 revolutions. The Los Angeles Coefficient is calculated from the proportion of the sample reduced to less than 1.6 mm in size.

After physical and mechanical characterization, leaching tests have been carried out on artificial aggregates. The aggregates have been submitted to leaching test for monolithic material according to [35], carried out with leachant renewals at 2 and 18 h and a total duration of 48 h ensuring a liquid volume/solid surface ratio of 10 cm. As in the granular material case, the pH must be brought to 2 by means of 1 M HNO_3_ before the metals released are analyzed. For environmentally critical systems, the cumulative leaching data after 72, 336 and 672 h have been determined.

## 3. Experimental Results and Discussion

### 3.1. Physico-Chemical Characterization of Residues

**Figure 3:**
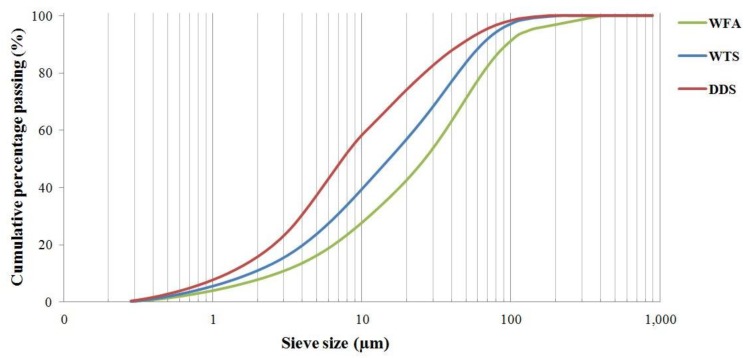
Particle size distributions of weathered coal fly ash, wastewater treatment sludge and desulfurization device sludge.

In Figure 3, particle size distributions of the three residues are reported. As far as the chemical composition is of concern, the three residues exhibit a high heterogeneity, as can be observed in Table 2.

DDS shows the highest water content and the maximum LOI. In the case of WFA, the high water content is related to the storage method. 

**Table 2:** Waste samples chemical compositions, wt %.

Analyte	Al_2_O_3_	Na_2_O	K_2_O	SO_3_	CaO	Fe_2_O_3_	MgO	MnO	P_2_O_5_	TiO_2_	SiO_2_	LOI
WFA	28.10	0.87	1.89	0.08	4.32	6.99	1.59	0.06	1.12	0.04	53.71	6.0
WTS	6.43	0.52	0.17	2.74	32.70	19.70	7.01	0.10	0.29	0.31	7.50	34.5
DDS	4.60	1.33	0.26	38.80	22.30	1.00	1.97	0.03	0.28	0.20	12.05	11.1

It is well known that coal fly ashes are essentially aluminosilicates which can behave as pozzolanic material, and so be able to substitute partially Portland cement. On the other hand, WTS main chemical components are calcium and iron oxides, with quite relevant amounts of SiO_2_, MgO, Al_2_O_3_, and SO_3_. Finally, DDS is characterized by the maximum content of SO_3_ among the wastes here studied. Apart from this, significant quantities of CaO, SiO_2_, and Al_2_O_3_ can be observed. In Table 3, the amount of heavy metals in the three wastes can be found, showing that the highest environmental risk is related to WTS.

**Table 3:** Heavy metals content in wastes samples, ppm.

Analyte	Cd	Cr	Cu	Ni	Pb	Se	V	Zn
**WFA**	10	40	30	50	20	10	110	50
**WTS**	10	60	100	80	10	10	160	1390
**DDS**	10	20	10	20	10	10	50	10

**Figure 4:**
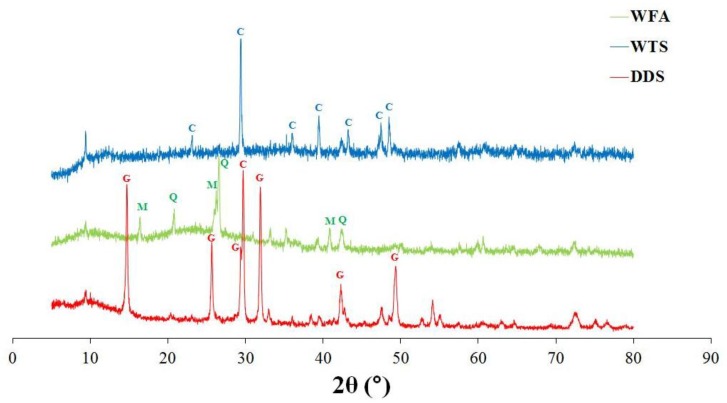
XRD analysis results for the three coal combustion residues (C = Calcite; M = Mullite; Q = Quartz; and G = Gypsum).

**Figure 5:**
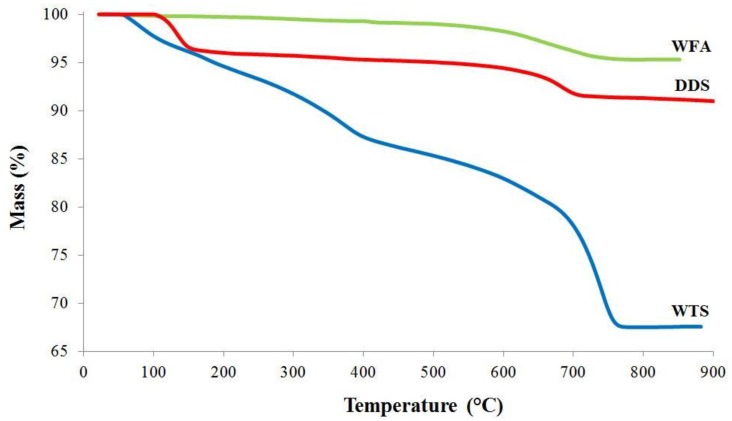
Thermal analysis results for the three coal combustion residues.

The results reported in Table 2 are in agreement with XRD and thermal analysis, which are reported in Figure 4 and Figure 5, respectively. WFA (green curve in Figure 4) is highly amorphous with the presence of a small amount of Quartz and Mullite. The WTS (blue in Figure 4) sample is mainly composed of calcite, while DDS (red in Figure 4) shows calcium sulfate hemihydrate and calcite peaks. 

Gravimetric thermal analysis, reported in Figure 5, confirms the results of XRD analysis in terms of identified phases. Observing the WFA curve, the range from 500 °C to 700 °C can be related to combustion of unburnt carbon in coal fly ash. As far as WTS curve, iron hydroxides decomposition between about 200 °C and 500 °C can be observed. For DDS, it can be argued that calcium sulfate hydrate decomposes in the range between about 100 °C and 150 °C. Considering both the WTS and DDS curves, in the range between about 700 °C and 800 °C the carbonates decomposition occurs with CO_2_ loss. The overall weight loss is 5%, 30%, and 8%, for the samples WFA, WTS, and DDS, respectively.

The leaching behavior of WFA, DDS, and WTS, for which few results can be found in literature [39], has been analyzed to evaluate the potential environmental issues related to the recycling of coal combustion wastes in artificial aggregates manufacturing. The release results are reported in Table 4. According to Italian regulation on simplified recovery procedure for non-hazardous wastes [40], the results obtained by applying the leaching test [35] must be compared to the concentration limits reported in Table 4. Particularly, the comparison must be carried out considering the release at three days. It can be observed that only Ni content poses a management issue, even if a few other metal (Cr, Cu, Se) contents are just below the limits. In the next section the leaching behavior on monolithic product will be evaluated, to ensure that also these small excesses of released metals will be immobilized by the cementitious binder systems.

**Table 4:** Metals release of samples: Weathered Fly Ash (WFA), Wastewater Treatment Sludge (WTS) and Desulfurization Device Sludge (DDS), μg/L.

Analyte	Cd	Cr	Cu	Ni	Pb	Se	V	Zn
**WFA**	0.04	8.37	13.15	13.71	0.31	1.23	0.35	0.67
**WTS**	0.10	26.22	34.76	74.38	0.12	7.10	0.01	3.51
**DDS**	0.57	4.37	7.67	10.98	0.23	4.37	12.25	2.15
Limits *	5	50	50	10	50	10	250	3000

* Ministerial Decree 5/4/2006 n. 186 [40].

### 3.2. Physical, Mechanical Properties, and Heavy Metals Release of Artificial Aggregates

The physical and mechanical properties of the cold bonded aggregates are influenced by both process and curing parameters and by raw materials hydration kinetics. In fact, as already pointed out in literature [36,41,42], the presence of chemical gypsum must be analyzed because the sulfate phases could give rise to ettringite formations. Thus, considering the simultaneous presence of a pozzolanic materials such as coal fly ash, a final heterogeneous hydration product containing both ettringite and calcium silicate hydrate (C–S–H) could be generated. In order to assess this particular hydration behavior, both chemical and thermal analyses have been carried out. 

**Figure 6:**
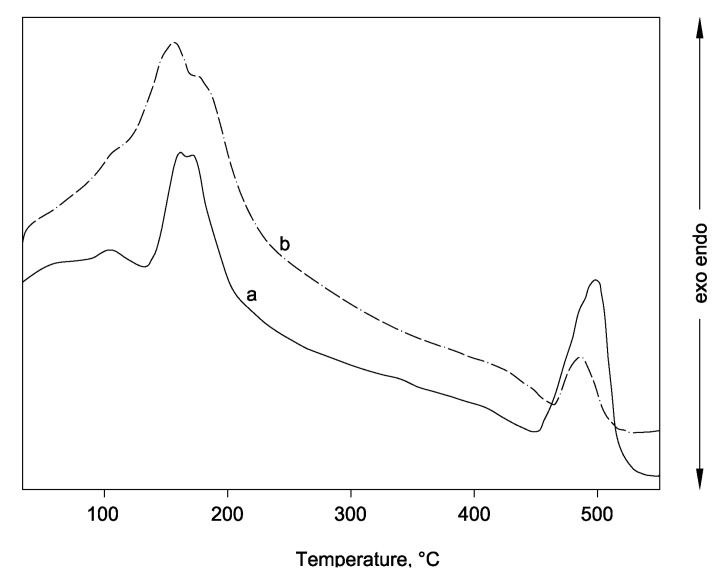
Thermal analysis results for the system C_40_D_30_ cured at 40 °C for (**a**) 7 days; and (**b**) 28 days.

**Figure 7:**
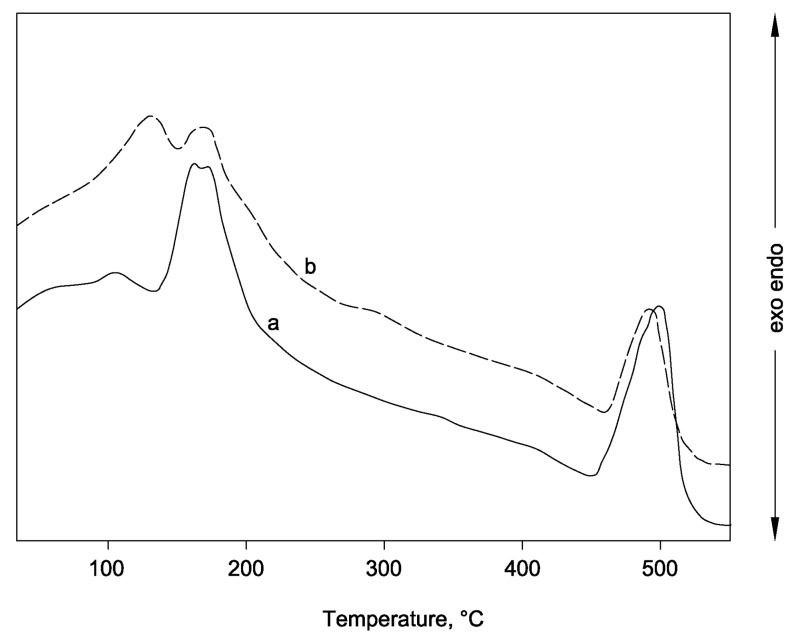
Thermal analysis results for the system C_40_D_30_ cured at room temperature for (**a**) 7 days; and (**b**) 28 days.

Figure 6 shows the two thermograms (curves a and b), obtained for the system C_40_D_30_ cured at 40 °C for 7 and 28 days, respectively. It can be seen that the endothermic effects, due to the decomposition of neo-formed hydrated phases, calcium silicate hydrate (C–S–H), and ettringite, and those due to the decomposition of unreacted gypsum and calcium hydroxide can be observed. Specifically, the endothermic peaks at 95 ± 25 °C, 125 ± 20 °C, 175 ± 15 °C and 495 ± 10 °C can be associated to C–S–H, ettringite, gypsum, and calcium hydroxide, respectively [36,41,42]. A very good conversion of raw materials towards ettringite can be detected in curve b, where the calcium hydroxide peak is widely reduced and the gypsum effect is almost completely included in the ettringite one. In this curve, a shoulder associated to the C–S–H formation is also present. The thermal effects shown in curve a of Figure 7 show that, at room temperature, hydration reactions happen more moderately. In fact, the presence of a higher amount of unreacted calcium hydroxide and gypsum is clearly evident.

The last results are confirmed by the XRD patterns for C_40_D_30_ system cured for 28 days at 40 °C (data not shown) for which the main crystalline phases observed are ettringite, gypsum, calcium hydroxide, mullite, and quartz. The other systems containing DDS, showed qualitatively similar behavior but, due to the differences in composition, the areas of endothermic peak were lower.

The complete set of above results confirm that binder mixtures containing two wastes, such as weathered coal fly ash and chemical gypsum, and calcium hydroxide can give hydrated system based on ettringite and calcium silicate hydrate. Furthermore, the same type of coal fly ash shows a good reactivity towards lime. This is in agreement with previous studies where aluminosilicates of higher quality were mixed with natural and chemical gypsum and lime [36,41,42].

**Figure 8:**
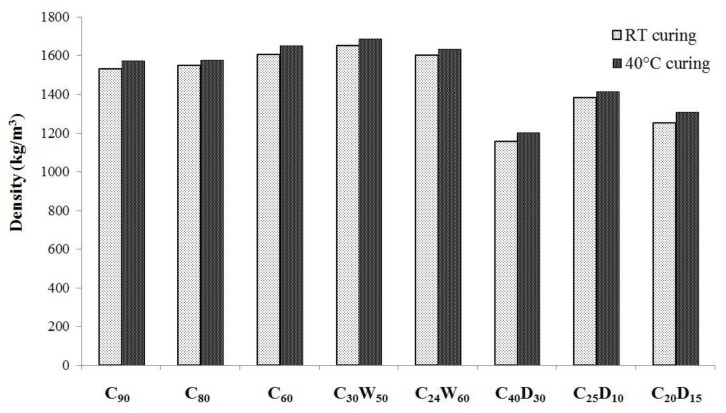
Dry density of artificial aggregates.

**Figure 9:**
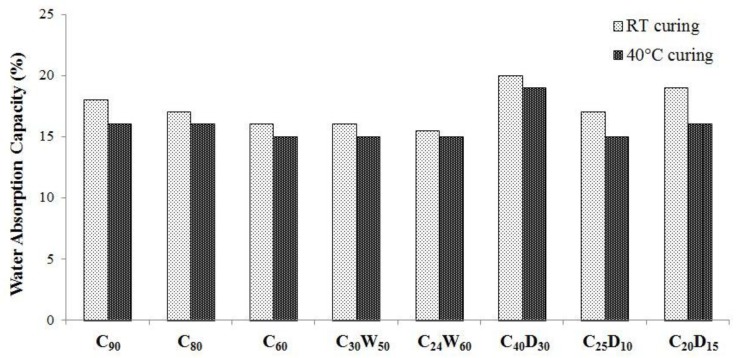
Water absorption capacity of artificial aggregates.

By means of the pelletization process, 6 ÷ 18 mm size aggregates have been obtained. In Figure 8 and Figure 9 the results of physical characterization are reported in terms of dry density and water absorption capacity. According to [38], the artificial aggregates can be classified as lightweight as dry density values are always lower than 2000 kg/m^3^. Aggregates containing DDS show an average dry density, which is about 20% lower than other ones. Particularly, C_60_ and C_30_W_50_ show the highest density for 40 °C and room temperature curing, respectively.

Absorption properties follow the overall trend of density for the various designed compositions. In fact, C_40_D_30_ aggregates, which do not show a very dense structure, behave as highly porous and reach the highest value of water absorption capacity (WAC). The WAC of all the hardened mixtures ranges from 15% to 20%, revealing to be quite similar to commercial lightweight aggregates.

**Figure 10:**
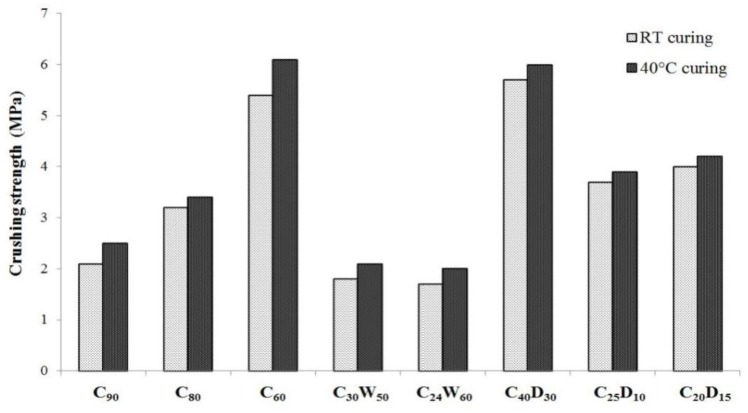
Crushing strength of artificial aggregates.

Crushing strength values are reported in Figure 10. These values are quite heterogeneous, except for the two mixtures that have been designed on a theoretical basis (*i.e.*, C_60_ and C_40_D_30_) rather than on economical and management considerations. In fact, going from C_90_ to C_60_, strength increases more than 100%. C_60_ mixture is designed with WFA/L ratio fixed at 3:2, while for C_80_ and C_90_ is 1:4 and 1:9, respectively.

C_30_W_50_ and C_24_W_60_ aggregates exhibit lower strength values than C_80_ and C_90_, due to the decrease of hydration products, which hinders the development of a compact microstructure. Finally, the mixtures, C_25_D_10_ and C_20_D_15_, seem to be a reliable compromise between the two needs of recovery different wastes and obtain satisfactory mechanical properties. In order to have a commercial benchmark, a widespread, lightweight expanded clay aggregate has been considered (*Leca^®^*). The crushing strength of this reference ranges from 0.7 to 4.5 MPa, depending on the size, so it can be argued that all the mixtures here presented could be suitable for many civil engineering applications where high performance isnot required.

**Figure 11:**
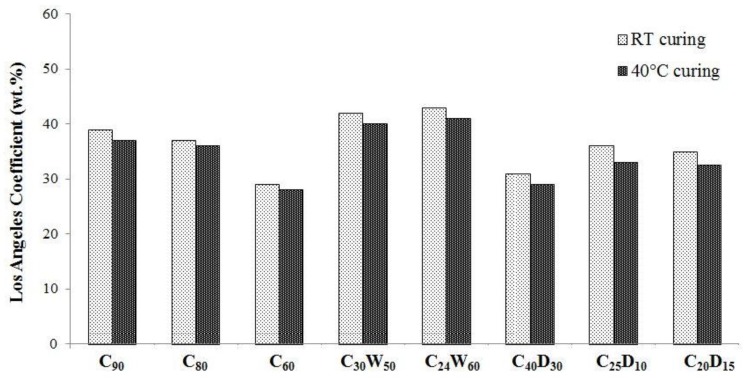
Los Angeles coefficient of artificial aggregates.

Los Angeles coefficients of produced aggregates are shown in Figure 11. Almost all tested samples have exhibited a *LA*_40_ fragmentation resistance class, according to table 9 of UNI EN 13242 [43]. As expected, a correlation between crushing and fragmentation resistance can be established. In fact, best mechanical performances are obtained for C_60_ and C_40_D_30_ aggregates. 

Considering all the values obtained in physico-mechanical testing of artificial aggregates, we can argue that a low temperature curing (40 °C) can improve, even if not so relevantly, hardened products performance.

After physico-mechanical characterization, stabilization properties of designed mixtures have been also evaluated. In Table 5, heavy metals release behavior of matrices has been reported for the elements Cr, Cu, Ni, and Se.

**Table 5:** Leaching behavior of the aggregates: Heavy metals release, µg/L.

Analyte		Cr			Cu			Ni			Se		
Renewal times, days		3	14	28	3	14	28	3	14	28	3	14	28
**C_30_W_50_**	**RT**	3.10	3.44	3.49	4.06	4.38	4.47	6.99	7.61	7.72	0.78	0.85	0.89
	**40 °C**	2.95	3.22	3.28	3.85	4.11	4.28	6.58	7.39	7.51	0.71	0.82	0.87
**C_24_W_60_**	**RT**	3.26	3.57	3.65	4.11	4.43	4.51	8.59	8.98	9.13	0.76	0.87	0.92
	**40 °C**	3.09	3.37	3.52	3.87	4.09	4.21	7.63	8.11	8.25	0.68	0.81	0.88
**C_25_D_10_**	**RT**	2.23	2.45	2.51	2.16	2.31	2.42	4.15	4.53	4.61	0.43	0.51	0.53
	**40 °C**	2.12	2.36	2.43	1.94	2.33	2.41	4.09	4.41	4.57	0.41	0.48	0.50
**C_20_D_15_**	**RT**	2.39	2.73	2.81	2.09	2.54	2.62	4.62	5.17	5.35	0.49	0.57	0.61
	**40 °C**	2.25	2.61	2.78	1.87	2.07	2.15	4.48	4.87	4.96	0.47	0.56	0.62

It is clear from Table 5 that 40 °C curing generally allows a better stabilization/solidification because temperature enhances hydration kinetics. Binding systems containing WFA and DDS give a better stabilization performance in comparison with C_30_W_50_ and C_24_W_60_. This observation can be explained by the differences in hydration products and, so, in microstructure. In fact, as will be clear in next section, C_25_D_10_ and C_20_D_15_ mixture hydration also produces ettringite, which improves the stabilizing capability of the system.

## 4. Conclusions

The main aim of this experimental program was the feasibility assessment of a sustainable process for the recycling of coal combustion solid residues, namely Weathered Fly Ash (WFA), Wastewater Treatment Sludge (WTS), and Desulfurization Device Sludge (DDS). WTS acts mostly as an inert component in binding mixtures, while DDS is able to give hydration expansive products such as ettringite. This is very important for mechanical performance and stabilizing properties of the matrices, because systems containing ettringite and C–S–H exhibit several improvements in comparison to the aggregates containing the unreactive waste, *i.e.*, WTS. The short term (three days) results of leaching test have been evaluated in order to assess the feasibility of the recycling process here studied according to Italian regulation on simplified recovery procedure for non-hazardous wastes, revealing that this process does not pose relevant environmental issues. Physical and mechanical properties of the obtained lightweight artificial aggregates, even if compared to those of market alternatives, demonstrated to be very interesting for a productive reuse of coal combustion residues, considering that only non-weathered and non-rejected fly ash has a common recycling pathway in blended Portland cements. It can be concluded that this preliminary phase of raw waste characterization and artificial aggregates manufacturing provides good results in order to establish the suitability of the here proposed recovery process. Nevertheless, a further experimental program must be carried out. Particularly, it could be very useful to manufacture lightweight concrete panels with these artificial aggregates, analyzing durability of both aggregates and concrete. This will be the subject of future studies.
